# The impact of reality shock on professional identity among junior nurses: a latent profile analysis

**DOI:** 10.3389/fmed.2026.1892812

**Published:** 2026-07-23

**Authors:** Xue Li, Xu Yan Liu, Yi Wei Li, Ping Zhou, Fan Jiang

**Affiliations:** 1 Department of Gynecology, Deyang People'Hospital, Deyang, China; 2 Department of Infectious Diseases, Deyang People'Hospital, Deyang, China

**Keywords:** reality shock, professional identity, junior nurses, latent profile analysis, influencing factors

## Abstract

**Background:**

Reality shock among junior nurses is a significant issue that reflects the gap between expectations and reality, potentially associated with their professional identity. However, the distribution of reality shock patterns across subgroups and its correlation with professional identity remain unclear.

**Objectives:**

To identify latent categories of reality shock among junior nurses using latent profile analysis, explore influencing factors of different profiles, and examine differences in professional identity across categories.

**Methods:**

A cross-sectional study was conducted from August to October 2025, enrolling 238 junior nurses ( ≤ 3 years of service) from a tertiary general hospital in Sichuan Province via convenience sampling. Data were collected using a demographic questionnaire, a reality shock scale, and a professional identity assessment scale. Latent profile analysis identified distinct reality shock categories; chi-square tests, independent-samples *t*-tests, and one-way ANOVA assessed demographic differences across categories; stratified linear regression determined the impact of reality shock on professional identity.

**Results:**

Two latent categories were identified: “low reality shock” (*n* = 143, 60.1%) and “high reality shock” (*n* = 95, 39.9%). Employment type, average weekly overtime frequency, reasons for choosing nursing, and job satisfaction were associated factors of reality shock subtypes (*P* < 0.05). After controlling for demographic variables, high levels of reality shock are negatively associated with professional identity; passive career choice, low job satisfaction, and lack of education were also significant negative predictors.

**Conclusion:**

Junior nurses' reality shock exhibits significant within-group heterogeneity, and high levels of reality shock are negatively associated with professional identity. Nursing managers and educators should prioritize identifying high-risk groups and promoting smooth role transition by optimizing workload management, strengthening professional identity education, and establishing a multidimensional support system.

## 1 Introduction

As the new generation of the nursing workforce, entry-level nurses' career development directly affects the quality of nursing care and patient safety (1, 2). With the increasing complexity of the healthcare environment and the diversification of patient needs, entry-level nurses face greater challenges as they transition from academic education to clinical practice (3, 4). Failure to adapt during this critical transition period not only affects nurses' job satisfaction and mental health but may also lead to burnout and a tendency to leave the profession, thereby threatening the stability of the nursing workforce and patient safety (5, 6). Therefore, addressing career adaptation among novice nurses, exploring its influencing factors, and developing effective intervention strategies are crucial for promoting the development of the nursing workforce and ensuring patient safety.

The concept of “Reality Shock” was first proposed by Kramer (7) in 1974. It refers to the psychological confusion and value conflicts experienced by new nurses upon entering clinical practice due to the gap between the idealized expectations formed through academic education and clinical reality. Work-related stress, complex interpersonal relationships, and professional expectations further exacerbate the issue of reality shock (8, 9). Approximately 60%−90% of novice nurses experience reality shock to varying degrees, with about 30%−40% experiencing moderate to severe reality shock (10, 11). Existing research indicates that high levels of reality shock not only increase stress, anxiety, and burnout among junior nurses but are also associated with decreased job satisfaction, increased turnover intentions, and impaired professional adaptation (12–14), thereby affecting the stability and healthy development of the nursing workforce. Furthermore, high levels of reality shock among nursing staff may increase the risk of care omissions and adverse events, adversely affecting nursing quality and patient safety (15).

Professional identity is a comprehensive reflection of an individual's cognitive understanding, emotional evaluation, and behavioral commitment to their professional role (16). For nurses, the formation of professional identity is a dynamic socialization process influenced by a combination of personal factors, organizational environment, and clinical experiences (17). Professional identity plays a crucial role in nurses' career development; nurses with high professional identity demonstrate greater work engagement, higher job satisfaction, and a lower tendency to leave the profession, which is particularly critical during the early stages of their careers (18–20). Junior nurses are currently in a critical phase of professional identity formation, and their experiences and adaptation during this period will have a profound impact on their long-term career development.

As medical standards and patient needs continue to evolve, nursing work has become increasingly challenging and uncertain. Based on Krame's (7) reality shock theory, nurses' professional identity is influenced by various factors, including individual characteristics, professional experiences, and the work environment. Among these, reality shock—a common negative occupational stress experience during the professional adaptation process of newly hired nurses—may significantly associated with professional identity. Against this backdrop, efforts to enhance professional identity among junior nurses should focus on their levels of reality shock and its associated factors. Previous studies have shown that nurses' reality shock is negatively correlated with variables such as professional values, self-efficacy, and role conflict (5, 15, 21). However, few studies have evaluated the impact of reality shock on the professional identity of junior nurses (22, 23), which overlooks this important group and the heterogeneity within it (24), hindering accurate assessment and targeted intervention.

Latent Profile Analysis (LPA) is a person-centered statistical method capable of identifying latent heterogeneous subgroups within a population based on response patterns of observed indicators (25). Compared to traditional cluster analysis, LPA is based on a probabilistic model, enabling more rigorous statistical inference and model fit assessment. In recent years, LPA has been widely applied in nursing research and has been successfully used to identify latent categories of nurse burnout, psychological distress, and occupational quality of life (26–28). Identifying latent categories of reality shock can facilitate a deeper understanding of the heterogeneity in the adaptation processes of junior nurses.

Based on Kramer's (7) theory of reality shock, reality shock has a potential impact on nurses' professional identity. Few previous studies have examined the impact of reality shock on professional identity among novice nurses, and existing research has largely overlooked the heterogeneity of this population. This study employs latent profile analysis to identify latent categories of reality shock among novice nurses and their associated factors, and to explore the relationship between different reality shock profiles and professional identity. The findings of this study will provide a reference for developing personalized support strategies to promote the development of professional identity among novice nurses.

## 2 Methods

### 2.1 Study design, setting, and participants

This study employed a cross-sectional design using convenience sampling. Between August and October 2025, 238 nurses from a tertiary general hospital in Sichuan Province, China, were selected to participate in this study. Inclusion criteria included: (1) registered nurses holding a nationally certified professional license; (2) with ≤ 3 years of work experience; (3) Voluntary participation in the study. Exclusion criteria: Nurses not on clinical duty due to various reasons (e.g., further training, maternity leave, vacation, health issues).

### 2.2 Data collection

The survey questionnaire was distributed and collected via the online platform “wenjuanxing” (http://www.wjx.cn). An introductory page provided participants with the study objectives and instructions for completion. After obtaining informed consent, participants could click a link to complete and submit the questionnaire independently. To enhance the quality of online data collection, each IP address was restricted to completing the questionnaire only once. Respondents could only successfully submit the questionnaire after completing all sections, thereby minimizing data missingness to the greatest extent possible.

### 2.3 Measurements

#### 2.3.1 General characteristics of participants

These included gender, marital status, highest level of education, place of residence, whether an only child, professional title, employment type, average monthly income, average number of night shifts per month, average frequency of overtime per week, reasons for choosing a nursing career, and satisfaction with the nursing profession.

#### 2.3.2 Reality shock scale (RSS)

The Reality Shock Scale was developed by Turkish scholars Çiriş Yildiz et al. (29) and localized into Chinese by Wu et al. (30). It comprises four dimensions—Relationships and Cooperation (21 items), Professional Knowledge (11 items), Competence (6 items), and Work Performance (3 items)—for a total of 41 items. It employs a 5-point Likert scale, ranging from “Never” to “Always” (scored 1–5). The total score ranges from 41 to 205 points; a higher score indicates a greater degree of reality shock experienced by newly hired nurses. The scale demonstrates good internal consistency and reliability; in this study, Cronbach's α was 0.960, and the S-CVI was 0.961.

#### 2.3.3 Professional identity scale

The Nurse Professional Identity Assessment Scale, developed by Liu (31), was used. This scale comprises five dimensions: professional cognitive evaluation (9 items), professional social skills (6 items), professional social support (6 items), coping with professional setbacks (6 items), and professional self-reflection (3 items), totaling 30 items. The scale uses a 5-point Likert scale ranging from “strongly disagree” to “strongly agree,” with scores ranging from 1 to 5 for each item. The total score ranges from 30 to 150, with higher scores indicating a higher level of professional identity. In this study, the Cronbach's alpha coefficient for the scale was 0.938, with Cronbach's alpha coefficients for each dimension exceeding 0.7. The Kaiser-Meyer-Olkin (KMO) value for construct validity was 0.949, indicating that the scale possesses good reliability and validity (32).

### 2.4 Statistical analysis

Mplus version 8.3 (Muthén & Muthén, Los Angeles, CA, USA) was used to explore the latent categories of reality shock among junior nurses. The LPA was conducted using the scores of all 41 items of the Reality Shock Scale as indicators. These 41 items span four dimensions: Relationships and Cooperation (items 1–21), Professional Knowledge (items 22–32), Competence (items 33–38), and Work Performance (items 39–41). Starting from the initial model (1 profile), we sequentially explored 1 to 3 latent profile models until the most appropriate model was identified via the log-likelihood test. Model fit indices for the LPA model included the Akaike Information Criterion (AIC), the Bayesian Information Criterion (BIC), and the adjusted Bayesian Information Criterion (aBIC); lower values indicate better model fit. Classification accuracy was assessed using entropy (ranging from 0 to 1, with values closer to 1 indicating better performance). The Lo-Mendell-Rubin (LMR) test and the Bootstrap Likelihood Ratio Test (BLRT) were used to evaluate the *p*-values for comparing models with different numbers of latent categories. A low *p*-value indicates that the k-class model fits better than the k-1-class model. IBM SPSS Statistics Version 27.0 (IBM Corporation, Armonk, NY, USA) was used to analyze differences in demographic characteristics and work-related information across different latent categories using the chi-square (χ^2^) test. Independent-samples *t*-tests or One-way analysis of variance (ANOVA) were used to compare the differences in demographic characteristics and work-related information of junior nurses with their professional identity. Furthermore, hierarchical linear regression was employed to determine the impact of reality shock on professional identity. *P* < 0.05 was considered statistically significant (two-tailed).

### 2.5 Ethical considerations

This study adhered to the principles of the Declaration of Helsinki. The study was approved by the Ethics Committee of Deyang People's Hospital (Date: February 12, 2025 / No.: 2025-04-012-K01), and informed consent was obtained from all participants.

## 3 Results

### 3.1 Participant characteristics

**Figure 1:**
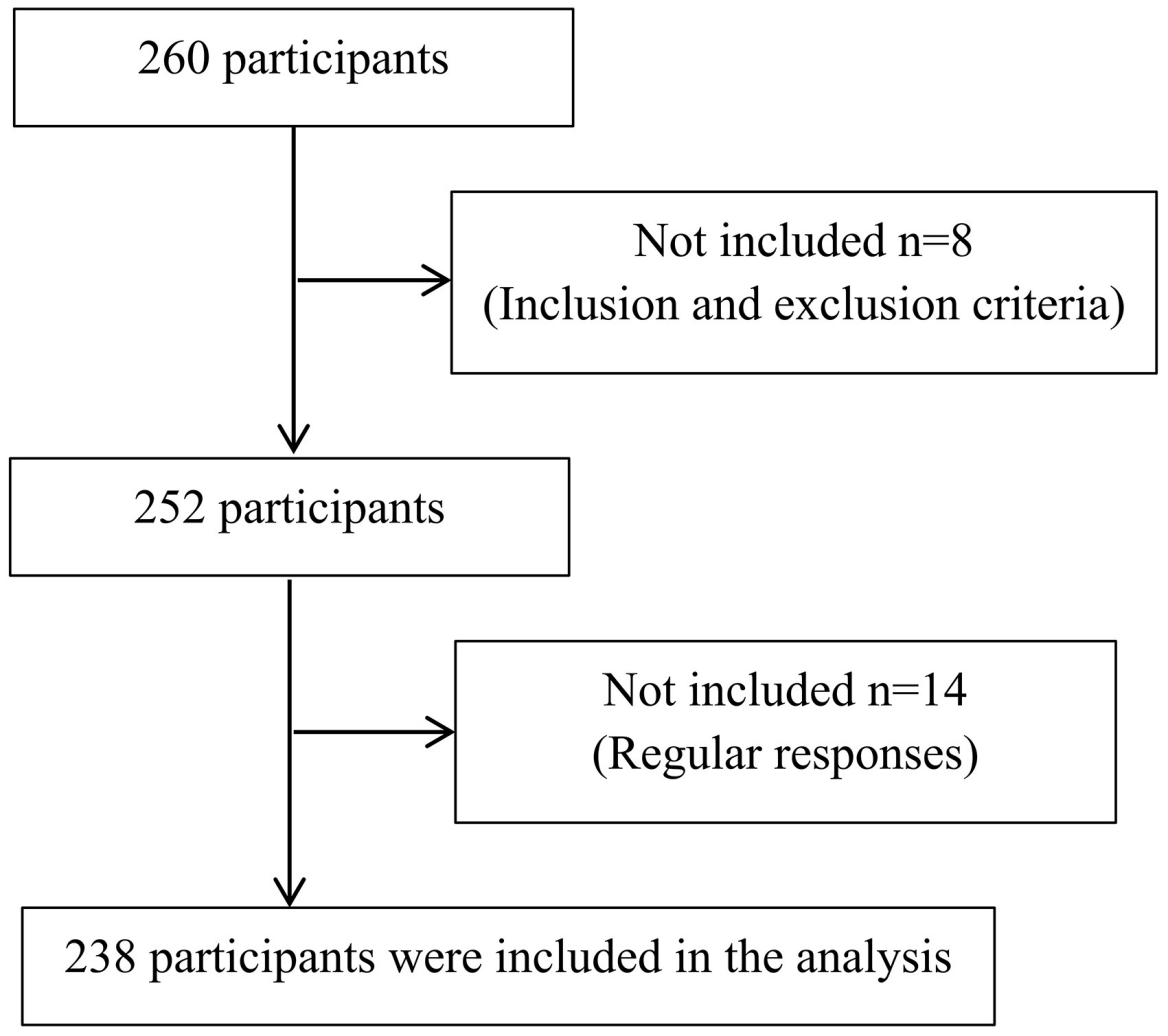
Flow diagram of participant recruitment and selection.

A total of 260 junior nurses participated in this study. After excluding 22 invalid questionnaires, 238 questionnaires were included in the statistical analysis. The vast majority of the study population was female (88.2%), while males accounted for only 11.8%. Most nurses were unmarried (73.9%), while only 26.1% were married. Additionally, the majority held a bachelor's degree or higher (70.6%), and 29.4% held a junior college diploma. Most held the professional title of “nurse” (74.8%), while only 21.4% were “senior nurses” and 3.8% were “ nurse-in-charge.” The flowchart for this study is shown in Figure 1.

### 3.2 Analysis of potential profiles of reality shock impact

To identify the optimal model, this study estimated three models separately; Table 1 displays the fit statistics for each model. The results show that when moving from a 1-class to a 2-class model, AIC, BIC, and ABIC all decreased significantly, indicating a marked improvement in model fit. Additionally, the LMR test (*P* = 0.0011) and BLRT test (*P* = 0.000) for the two-class model reached statistical significance, and the entropy value was as high as 0.998, indicating excellent classification accuracy. Although AIC, BIC, and ABIC further decreased slightly in the three-class model, its LMR test (*P* = 0.7306) did not reach statistical significance, indicating no statistically significant improvement compared to the two-class model. The average posterior classification probability (AvePP) for Model 2 is as follows: The AvePP for Class 1 (low reality shock) is 1.000, and the AvePP for Class 2 (high reality shock) is 0.999. The modal assignment approach is appropriate and widely accepted under such conditions. Therefore, taking into account the proportion of each model and the practical significance of the results, this study adopts the two-class model for subsequent interpretation and analysis.

**Table 1:** Impact of reality shock data on the fitting metrics of potential profile models.

Model	AIC	BIC	ABIC	*P*		Entropy	Group size
				LMR	BLRT		
1	32,115.807	32,400.533	32,140.619				238
2	25,231.891	25,662.453	25,269.412	0.001	0.000	0.998	143/0.60, 95/0.40
3	22,559.051	23,135.448	22,609.28	0.4199	0.000	0.996	132/0.55, 72/0.30, 34/0.14

AIC, Akaike information criterion; BIC, Bayesian information criterion; ABIC, sample-adjusted Bayesian information criterion; LMR, Lo-Mendell-Rubin corrected likelihood ratio test; BLRT, bootstrap likelihood ratio test.

**Figure 2:**
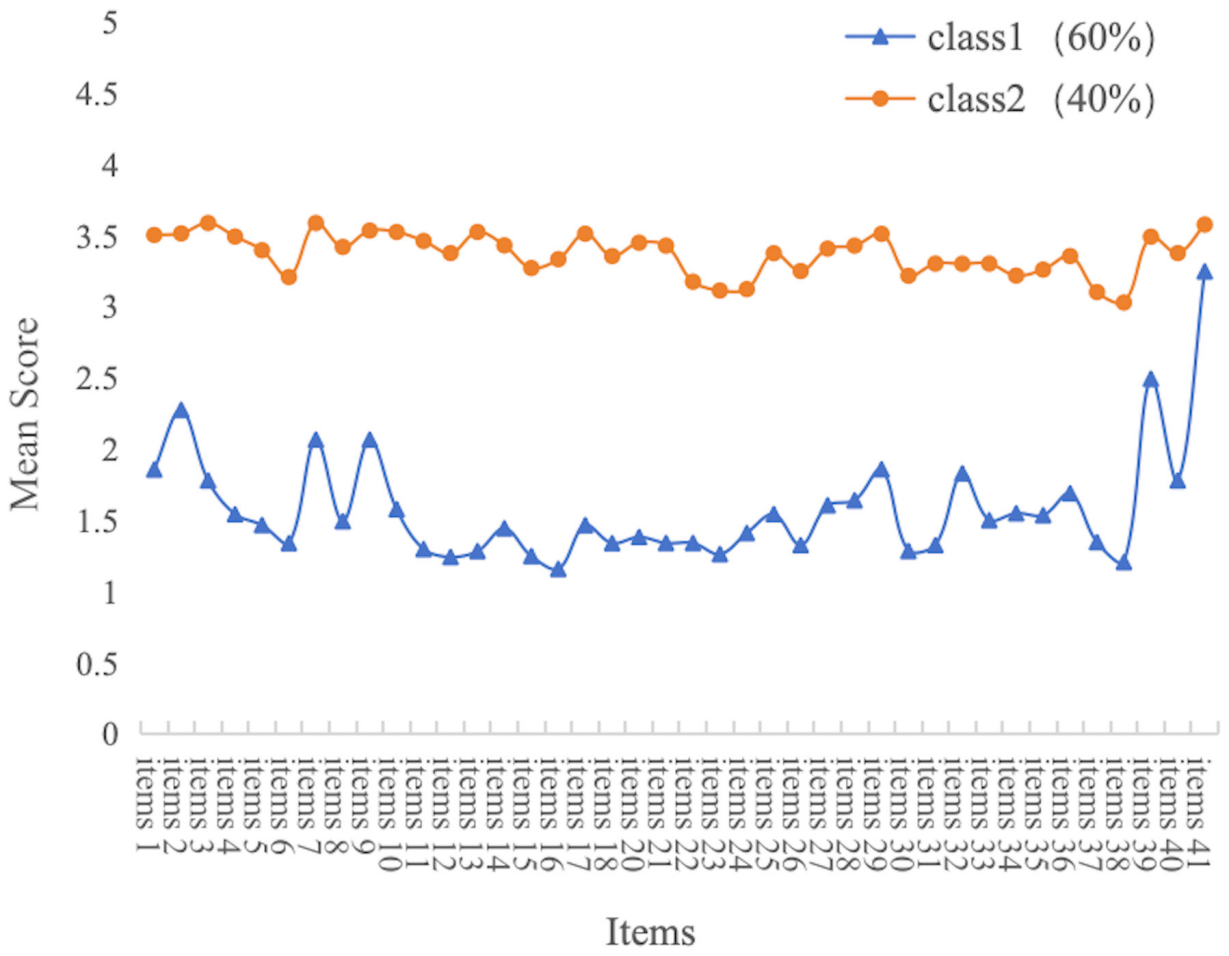
Scores of model 2 on 41 reality shock impact items. Relationship and collaboration dimension items (1–21), professional knowledge dimension items (22–32), competency dimension items (33–38), and work performance dimension items (39–41).

Table 2 shows the distribution of the two-class model across the four dimensions of reality shock, and Figure 2 displays the scores for the 41 items in the two-class model. Category 1 scored lower than Category 2 across all dimensions and was named the “low reality shock group,” comprising 143 cases (60%); Category 2, with scores at a higher level across all dimensions, was named the “high reality shock group,” comprising 95 cases, accounting for 40%.

**Table 2:** Distribution of reality shock impacts in the 2-profile model (*n* = 238).

Dimensions	Total sample (*n* = 238) M±SD	Class 1 (*n* = 143) M±SD	Class 2 (*n* = 95) M±SD	*t*	*P*
Relationships and cooperation	2.29 ± 1.11	1.52 ± 0.44	3.44 ± 0.78	−21.733	< 0.001
Professional knowledge	2.21 ± 1.1	1.5 ± 0.48	3.29 ± 0.86	−18.657	< 0.001
Competence	2.17 ± 1.14	1.47 ± 0.55	3.21 ± 0.99	−15.633	< 0.001
Work performance	2.9 ± 0.95	2.51 ± 0.78	3.48 ± 0.9	−8.882	< 0.001
Total score	94.06 ± 42.61	64.76 ± 15.19	138.17 ± 30.94	−21.472	< 0.001

M = mean, SD = standard deviation.

### 3.3 Results of univariate analysis of potential categories of reality shock among junior nurses

Table 3 presents the comparison results of the two potential categories based on general demographic data. Statistically significant differences (*P* < 0.05) were observed among junior nurses in different categories regarding employment type, average weekly overtime frequency, reasons for choosing nursing, and satisfaction with the nursing profession.

**Table 3:** Univariate analysis of factors affecting the potential categories of reality shock among junior nurses.

Variable	Total sample size (*n* = 238)	Class 1 (*n* = 143)	Class 2(*n* = 95)	*χ2*	*P*
	N (%)	n (%)	n (%)		
Gender				0.005	0.942
Female	210 (88.2%)	126 (88.1%)	84 (88.4%)		
Male	28 (11.8%)	17 (11.9%)	11 (11.6%)		
Highest level of education				0.358	0.550
Junior college diploma	70 (29.4%)	40 (28.0%)	30 (31.6%)		
Bachelor's degree or higher	168 (70.6%)	103 (72.0%)	65 (68.4%)		
Place of residence				0.027	0.870
Local	174 (73.1%)	104 (72.7%)	70 (73.7%)		
Non-local	64 (26.9%)	39 (27.3%)	25 (26.3%)		
Are you an only child?				0.375	0.541
Yes	111 (46.6%)	69 (48.3%)	42 (44.2%)		
No	127 (53.4%)	74 (51.7%)	53 (55.8%)		
Professional title				2.6	0.272
Nurse	178 (74.8%)	112 (78.3%)	66 (69.5%)		
Senior nurses	51 (21.4%)	27 (18.9%)	24 (25.3%)		
Nurse-in-charge	9 (3.8%)	4 (2.8%)	5 (5.3%)		
Marital Status				1.644	0.200
Married	62 (26.1%)	33 (23.1%)	29 (30.5%)		
Unmarried	176 (73.9%)	110 (76.9%)	66 (69.5%)		
Employment type				–	0.024^*^
Permanent staff nurses	4 (1.7%)	0 (0.0%)	4 (4.2%)		
Contract nurses	234 (98.3%)	143 (100.0%)	91 (95.8%)		
Monthly income				0.307	0.959
Less than 3,000 yuan	29 (12.2%)	17 (11.9%)	12 (12.6%)		
3,001–5,000yuan	173 (72.7%)	105 (73.4%)	68 (71.6%)		
5,001–7,000yuan	30 (12.6%)	17 (11.9%)	13 (13.7%)		
7,001 yuan or more	6 (2.5%)	4 (2.8%)	2 (2.1%)		
Physical condition				4.702	0.095
Poor	9 (3.8%)	6 (4.2%)	3 (3.2%)		
Generally	100 (42.0%)	52 (36.4%)	48 (50.5%)		
Good	129 (54.2%)	85 (59.4%)	44 (46.3%)		
Average number of night shifts per month				0.834	0.841
Less than 3 days	40 (16.8%)	22 (15.4%)	18 (18.9%)		
3–5 days	148 (62.2%)	89 (62.2%)	59 (62.1%)		
6–9 days	40 (16.8%)	26 (18.2%)	14 (14.7%)		
10 days or more	10 (4.2%)	6 (4.2%)	4 (4.2%)		
Average weekly overtime frequency				8.363	0.015^*^
Hardly	35 (14.7%)	25(17.5%)	10 (10.5%)		
Sometimes	184 (77.3%)	112(78.3%)	72 (75.8%)		
Frequently	19 (8.0%)	6(4.2%)	13 (13.7%)		
Have you been providing clinical care independently?				0.118	0.731
Yes	136 (57.1%)	83 (58.0%)	53 (55.8%)		
No	102 (42.9%)	60 (42.0%)	42 (44.2%)		
Reasons for choosing nursing				7.025	0.008^*^
Proactive	76 (31.9%)	55 (38.5%)	21 (22.1%)		
Passive	162 (68.1%)	88 (61.5%)	74 (77.9%)		
Job satisfaction				14.839	0.002^*^
Very satisfied	64 (26.9%)	48 (33.6%)	16 (16.8%)		
Fairly satisfied	103 (43.3%)	64 (44.8%)	39 (41.1%)		
Generally satisfied	64 (26.9%)	29 (20.3%)	35 (36.8%)		
Not very satisfied	7 (2.9%)	2 (1.4%)	5 (5.3%)		
Does your family support your work?				-	0.158
Yes	236 (99.2%)	143 (100.0%)	93 (97.9%)		
No	2 (0.8%)	0 (0.0%)	2 (2.1%)		
Did you receive any training at school on topics such as career identity and stress management?				0.478	0.489
Yes	219 (92.0%)	133 (93.0%)	86 (90.5%)		
No	19 (8.0%)	10 (7.0%)	9 (9.5%)		

^*^P < 0.05. Fisher's exact test was applied for variables with expected cell frequencies < 5.

### 3.4 Univariate analysis of professional identity among junior nurses

Independent-samples *t*-tests or one-way ANOVA were conducted on professional identity using general demographic data. The results showed statistically significant differences in professional title, reasons for choosing nursing, family support for work, satisfaction with the nursing profession, and whether they received relevant education on professional identity and stress coping during their academic training (*P* < 0.05). Given the extremely small number of permanent staff nurses (*n* = 4), chi-square assumptions were violated for employment type; Fisher's exact test confirmed the association (*P* = 0.024), though this finding should be interpreted with caution. (see Table 4).

**Table 4:** A comparison of professional identity among junior nurses based on demographic characteristics.

Variables	Category	*n*	%	M±SD	*P*
Gender	Female	210	88.2	120.26 ± 18.55	0.090
	Male	28	11.8	126.64 ± 19.50	
Marital status	Married	62	26.1	117.13 ± 19.81	0.058
	Unmarried	176	73.9	122.38 ± 18.21	
Highest level of education	Junior college diploma	70	29.4	120.66 ± 19.78	0.852
	Bachelor's degree or higher	168	70.6	121.15 ± 18.34	
Professional title	Nurse	178	74.8	122.60 ± 17.88	0.038^*^
	Senior nurses	51	21.4	115.08 ± 20.33	
	Nurse-in-charge	9	3.8	123.22 ± 21.42	
Employment type	Permanent staff nurses	4	1.7	107.25 ± 30.93	0.139
	Contract nurses	234	98.3	121.24 ± 18.48	
Monthly income	Less than 3,000 yuan	29	12.2	125.79 ± 19.82	0.322
	3,001–5,000 yuan	173	72.7	119.66 ± 18.34	
	5,001–7,000 yuan	30	12.6	123.70 ± 18.98	
	7,001 yuan or more	6	2.5	123.33 ± 23.17	
Reasons for choosing nursing	Proactive	76	31.9	133.24 ± 16.10	0.000^**^
	Passive	162	68.1	115.27 ± 17.10	
Does your family support your work?	Yes	236	99.2	121.23 ± 18.61	0.048^*^
	No	2	0.8	95.00 ± 21.21	
Job satisfaction	Very satisfied	64	26.9	138.33 ± 15.15	0.000^**^
	Fairly satisfied	103	43.3	117.38 ± 13.35	
	Generally satisfied	64	26.9	111.63 ± 18.02	
	Not very satisfied	7	2.9	101.86 ± 15.18	
Did you receive any training at school on topics such as career identity and stress management?	Yes	219	92	122.16 ± 18.06	0.001^**^
	No	19	8	107.74 ± 21.76	

^*^P < 0.05, ^**^P < 0.001.

### 3.5 Regression analysis of factors influencing professional identity

Hierarchical regression analysis was used to further explore the effects of various factors on professional identity. Model 1 included only general demographic variables, while Model 2 added the classification of real-life stressors to this foundation. The results showed (see Table 5): Model 1 explained 37.3% of the variance in professional identity (adjusted *R*^2^ = 0.373, *p* < 0.001). Among these, passive choice of nursing as a career (β = −0.142, *p* = 0.027), low job satisfaction (β = −0.461 to −0.265, all *p* < 0.001), and lack of professional identity education (β = −0.119, *p* = 0.025) were significant negative predictors of professional identity. In Model 2, after incorporating reality shock, the model's explanatory power increased to 38.7% (adjusted *R*^2^ = 0.387). The results showed that reality shock of Class 2 (Beta = −0.133, *p* = 0.013) was also a significant negative predictor of professional identity, and the aforementioned variables remained significant. In summary, the professional identity of junior nurses is influenced by multiple factors, among which the type of reality shock (Class 2) is an important predictor independent of demographic variables. Multicollinearity diagnostics showed that all variance inflation factors (VIF) were below 3.0 (range: 1.075–2.314), indicating no substantial multicollinearity.

**Table 5:** The impact of potential patterns of different reality shocks on professional identity in hierarchical linear regression.

Independent variables	Model1		95% CI	Model 2		95% CI
	Beta	P		Beta	P	
Professional title (ref: nurse)						
Senior nurses	−0.067	0.219	−7.886~1.819	−0.062	0.250	−7.607 ~1.993
Nurse-in-charge	0.036	0.506	−6.889~13.932	0.041	0.438	−6.243~14.357
Reasons for choosing nursing (ref: proactive)						
Passive	−0.151	0.020	−11.124~-0.958	−0.142	0.027	−10.728~-0.664
Does your family support your work? (ref:yes)						
No	−0.094	0.081	−40.714~2.402	−0.083	0.117	−38.434~4.316
Job satisfaction (ref: very satisfied)						
Fairly satisfied	−0.465	0.000	−22.885~-12.239	−0.452	0.000	−22.354~-11.801
Generally satisfied	−0.487	0.000	−26.994~-14.114	−0.461	0.000	−25.853~-12.998
Not very satisfied	−0.284	0.000	−43.486~-19.373	−0.265	0.000	−41.331~-17.260
Did you receive any training at school on topics such as career identity and stress management? (ref:yes)						
no	−0.119	0.026	−15.477~-0.975	−0.119	0.025	−15.350~-1.013
Reality Shocks (ref: class 1)						
Class 2				−0.133	0.013	−9.056 ~-1.100
*R^2^*	0.394	0.000		0.410	0.000	
ΔR^2^	0.373			0.387		
*F*	18.616			6.327		

## 4 Discussion

### 4.1 Latent profiles of reality shock among junior nurses

This study employed latent profile analysis to identify distinct categories of reality shock among junior nurses, namely the “low reality shock” group and the “high reality shock” group. This classification reflects the heterogeneity of reality shock among junior nurses across each latent dimension, complementing previous studies that treated junior nurses as a homogeneous group, and provides a basis for developing targeted interventions to reduce reality shock among junior nurses. The “low reality shock” group accounted for 60% of the sample (*n* = 143), with a total average reality shock score of (64.76±15.19) points. This group exhibited relatively low levels of reality shock, demonstrating the ability to gradually adapt to the clinical environment during the early stages of employment. They reported relatively weak perceptions of the gap between reality and expectations regarding job requirements, team collaboration, and professional competence. However, this group should not be overlooked; measures must be taken to prevent their reality shock from escalating to a more severe state. The “high reality shock” group accounted for 40% (*n* = 95) of the sample, a relatively high proportion, with a total average reality shock score of (138.17±30.94) points. This indicates that the incidence of reality shock is relatively high among junior nurses, which is consistent with previous research findings (15, 33). Particularly in the context of short tenure and insufficient clinical experience, reality shock is more likely to become a significant factor hindering their smooth transition into their new roles (10, 11).

Furthermore, the“high reality shock” group scored higher than the “low reality shock” group across all four dimensions, suggesting that reality shock among junior nurses manifests primarily as a difference in degree rather than a structural difference. This indicates that reality shock among junior nurses may represent a global adaptation difficulty rather than one confined to a single dimension. This finding aligns with Çiriş Yildiz et al.'s (29) view that reality shock among newly hired nurses exhibits multidimensional characteristics. Therefore, interventions addressing reality shock should not target a single dimension but rather adopt comprehensive strategies that simultaneously address multiple aspects, including interpersonal adaptation, the translation of professional knowledge, the development of competence, and the improvement of work performance. Given that the “Relationships and Collaboration” dimension had the most items and the highest scores, this suggests that interpersonal challenges may be the most prominent aspect of reality shock among junior nurses. This finding aligns with Kramer's (7) reality shock theory, which explicitly identifies interpersonal adaptation challenges as one of the most central sources of shock during the early transition phase. Multiple studies have also shown that inadequate interpersonal communication skills are a direct challenge faced by junior nurses (34), and difficulties in establishing nurse-patient relationships and integrating into the team also lead to interpersonal adaptation difficulties for junior nurses (35, 36). Therefore, clinical instructors and managers should pay particular attention to the adaptation difficulties new nurses face in cross-level communication, team collaboration, and nurse-patient relationships. Additionally, even within the “low reality shock” group, scores on the work performance dimension were relatively high. This suggests that junior nurses generally lack confidence in their work performance, and this self-doubt may be one of the most central and universal manifestations of reality shock. This is consistent with Duchscher's (3) description of role adjustment difficulties among junior nurses in his theory of transition shock. Future interventions should prioritize enhancing junior nurses' confidence in their work performance. Managers can establish standardized competency feedback mechanisms to help new nurses objectively assess their abilities through regular evaluations and personalized feedback; simultaneously, they should implement structured mentoring and scenario-based simulation training to allow nurses to accumulate clinical decision-making experience in a safe environment and gradually build professional self-efficacy; Furthermore, reflective practice activities such as group case discussions can be organized at the department level to guide new nurses in objectively attributing their work performance. This helps prevent the misinterpretation of normal learning curves as competency deficits, thereby effectively mitigating the real-world impact on work performance.

### 4.2 Analysis of factors influencing the potential dimensions of reality shock among junior nurses

Based on Kramer's (7) theory of reality shock, reality shock is influenced by multiple factors, including individual characteristics, the organizational environment, and professional socialization. Among demographic variables, the factors influencing the potential categories of reality shock primarily include employment type, average weekly frequency of overtime, reasons for choosing nursing, and satisfaction with the nursing profession. Differences in career expectations and role demands may underlie these distinct employment types. Compared to contract nurses, permanent staff nurses may have higher expectations regarding career development, job stability, and self-actualization; when a gap emerges between clinical reality and career expectations, they are more likely to experience a pronounced sense of psychological imbalance. This study found that junior nurses with a higher average weekly frequency of overtime work were more likely to be classified into the “high reality shock” group. Frequent overtime implies a heavy workload; overwork not only increases physical exhaustion and emotional burnout but also diminishes their sense of control over work pace, task requirements, and job competence, thereby intensifying negative experiences in clinical work. For junior nurses, due to their limited proficiency in the job and low level of integration into the team, their work efficiency is relatively low, making it difficult for them to handle heavy nursing tasks with ease. Excessive workloads also reduce their opportunities for learning, reflection, and adjustment, causing reality shock to accumulate and intensify more easily (37). We also observed that those who passively chose the nursing profession were more likely to be classified into the “high reality shock” group. This may be attributed to the fact that individuals who enter nursing passively lack stable career interests, clear career goals, and strong intrinsic motivation; when faced with high-intensity work and complex clinical environments, they are more likely to perceive a career gap and experience psychological imbalance (38). This is also supported by previous research, which indicates that new nurses who lack sufficient psychological and emotional support during the career transition period are more likely to experience adaptation difficulties and negative experiences under the pressure of role transition (39). Furthermore, those with lower job satisfaction in nursing were more prevalent in the “high reality shock” group. This suggests that persistent discrepancies between expectations and reality weaken junior nurses' positive perceptions of the nursing profession, while lower job satisfaction further exacerbates their subjective experience of reality shock. This finding aligns with previous research indicating a negative correlation between job satisfaction and reality shock (40).

Based on the above analysis, nursing managers should recognize the heterogeneity of reality shock among junior nurses and focus on permanent staff who frequently work overtime, entered the nursing profession passively, or have low job satisfaction. Early identification and targeted interventions should be implemented for high-risk groups. Nursing managers should establish a tiered and categorized system of targeted interventions tailored to the heterogeneity of reality shock among junior nurses. For permanent staff, role competence and a sense of belonging should be enhanced through strengthened mentor support (41) and progressive clinical empowerment (42); for nurses with frequent overtime, optimize human resource allocation through scheduling systems (43) and mandate compensatory time off and delayed rest periods following overtime (44) to reduce occupational fatigue and safety risks; for individuals who entered the nursing profession passively, provide early-career professional identity reshaping and career planning counseling (45), leveraging peer role models and the accumulation of successful experiences (46) to strengthen their intrinsic motivation and professional commitment; For those with low job satisfaction, establish regular post-hiring satisfaction monitoring combined with supportive group interventions (42) to alleviate emotional exhaustion and improve job satisfaction, thereby achieving a closed-loop management system—from early identification to targeted intervention—among high-risk populations.

### 4.3 The association between different latent profiles of reality shock on professional identity

Stratified regression analysis indicates that different categories of latent profiles of reality shock are significant associated with professional identity. After controlling for general demographic variables, the level of professional identity in the “high reality shock” group remained significantly lower than that in the “low reality shock” group. The model's explanatory power increased, and the statistical effect was significant, suggesting that reality shock has a significant negative impact on professional identity. The relatively small incremental explanatory power may be related to the fact that this study treated reality shock as a dichotomous variable. Compared to continuous variables, this approach may result in some loss of information; simultaneously, variables such as job satisfaction already explained a large proportion of the variance in professional identity, thereby limiting the additional explanatory power of reality shock. Despite the small increment, the independent and statistically significant association of reality shock with professional identity has practical relevance, as it suggests that interventions targeting reality shock may contribute to improving professional identity even after accounting for demographic and career-motivational factors. This result further supports the negative association between reality shock and professional identity and is consistent with the conclusions of previous studies (20, 47). Based on Kramer's (7) reality shock theory, the impact of reality shock on professional identity can be explained from the following perspectives. First, high reality shock weakens the professional ideals and value identification formed by junior nurses during their nursing education, thereby undermining their positive expectations regarding the nursing profession (19). Second, repeated frustrations and experiences of uncertainty in clinical practice may reduce their professional self-efficacy, causing them to doubt their ability to perform nursing duties (20). Furthermore, role conflict and role ambiguity can disrupt the process of professional role integration; when new nurses struggle to reconcile their ideal role expectations with their actual work experiences, the formation of professional identity is easily hindered (21). In this study, the “high reality shock” group represents nurses who are still in the stage of adaptation difficulties and have not yet completed a smooth transition. During this period, the foundation of professional identity is not yet solid; if timely support is lacking, the negative impact of reality shock may persist and even affect their willingness to remain in the profession.

Concurrently, passive career choice, low job satisfaction, and a lack of education related to professional identity were all significant negative predictors of professional identity. These findings indicate that the development of professional identity among junior nurses is influenced not only by reality shock but also by the combined effects of multiple factors, including career choice motivation, professional experience, and prior educational preparation. Based on this, nursing educators and administrators should focus their efforts simultaneously on both the student training phase and the initial transition to practice: on the one hand, strengthening guidance on professional values and education on professional identity to help individuals establish more stable intrinsic professional motivation; on the other hand, providing continuous support measures for junior nurses during the early stages of their careers, including stress management training, mentorship, and career development support. Particular attention should be paid to those at high risk of experiencing reality shock to facilitate their smooth transition into their professional roles and enhance their levels of professional identity.

## 5 Limitations

This study has the following limitations. First, the cross-sectional design does not allow for inferring a causal relationship between reality shock and professional identity, nor does it reveal the dynamic evolution of reality shock profiles over time. Future studies could adopt a longitudinal design, collecting data at multiple time points after new nurses begin their employment to examine the patterns of change in reality shock profiles and their dynamic impact on professional identity. Second, convenience sampling was used, and the sample was drawn solely from a single tertiary general hospital in Sichuan Province. Consequently, the sample's representativeness is limited, which may affect the generalizability of the results to other regions and hospitals of different levels. Future research should validate these findings in large-scale, multicenter, and multi-regional studies encompassing healthcare institutions of varying levels. Third, single-point self-report data introduces self-report and common method bias; future research should triangulate with supervisor ratings or objective performance indicators. Fourth, some categorical variables had very small cell sizes, introducing statistical instability; findings should be interpreted with caution. Future studies could also increase the sample size to enable more refined and clinically valuable identification of subgroups.

## 6 Conclusions

This study is the first to employ latent profile analysis to identify two latent categories of reality shock among junior nurses, revealing the heterogeneity of reality shock within this group. Employment type, frequency of overtime, reasons for choosing a nursing career, and job satisfaction were significant predictors of different latent profiles of reality shock. After controlling for general demographic variables, high reality shock remained a negatively associated factor of professional identity. The findings suggest that nursing administrators and educators should prioritize the group of junior nurses experiencing high reality shock, including those on permanent staff, those working frequent overtime, those who chose nursing passively, those with low job satisfaction, those who have not received education related to professional identity, as well as junior nurses who struggle with interpersonal adjustments and receive negative performance evaluations. Through comprehensive measures such as optimizing workload management, providing career planning guidance, strengthening professional identity education, and fostering a supportive work environment, these individuals can be helped to successfully navigate the critical period of role adaptation. This will mitigate the negative impact of reality shock on the development of professional identity, thereby promoting the stability and healthy development of the nursing workforce.

## Data Availability

The original contributions presented in the study are included in the article/supplementary material, further inquiries can be directed to the corresponding author.
